# Trends in Informal and Formal Long-Term Care Use Among Older Adults With Disabilities in Japan

**DOI:** 10.1093/geroni/igab046.1624

**Published:** 2021-12-17

**Authors:** Hidehiro Sugisawa, Yoko Sugihara, Erika Kobayashi, Taro Fukaya, Jersey Liang

**Affiliations:** 1 J.F. Oberlin University, Tokyo, Tokyo, Japan; 2 Tokyo Meropolitan University, Tokyo, Tokyo, Japan; 3 Tokyo Metropolitan Institute of Gerontology, Itabashi-ku, Tokyo, Japan; 4 Tokyo Metropolitan Institute of Gerontology, Tokyo, Tokyo, Japan; 5 School of Public Health, University of Michigan, Ann Arbor, Michigan, United States

## Abstract

Whether increased formal long-term care (LTC) reduces informal LTC use by serving as a substitute or has a complementary role that boosts both informal and formal LTC use has been an important issue for evaluating LTC policy effectiveness. We described trends in in-home LTC use among older adults and LTC availability in relation to changes in LTC policy in Japan. In addition, we examined whether these trends differ by living arrangements, gender, income, and disability levels. We used five waves of repeated cross-sectional data starting in 1999 to 2017. The use of both informal and formal LTC types combined increased until 2006 and then gradually decreased while remaining higher than in 1999. Although implementing the LTC program may have temporarily contributed to the complementary use of both LTC types, eligibility limitations brought about by LTC reform potentially reduced the effects of formal LTC’s complementary role.

